# Supplementary material for: Comparative single-cell analyses reveal evolutionary repurposing of a conserved gene programme in bat wing development
Source: Nat Ecol Evol. 2025 Jul 16;9(9):1626–42. doi: 10.1038/s41559-025-02780-x (PMC12420390; doi:10.1038/s41559-025-02780-x)
Supplement: Supplementary file 1 — Supplementary Tables 1–3. [file 41559_2025_2780_MOESM1_ESM.pdf]

nature ecology & evolution

Supplementary information https://doi.org/10.1038/s41559-025-02780-x

Comparative single-cell analyses reveal evolutionary repurposing of a conserved gene programme in bat wing development

In the format provided by the authors and unedited

Supplementary Tables

Supplementary Table 1.

Cell lines. This table contains wildtype and mutant mouse embryonic stem cell lines used and generated in this study and sgRNA sequences and mm39 coordinates of knock-in site at H11 locus.

ESC Line	*Source*	*Parental ESC Line*	*Type*	*sgRNA / Homology Construct*	*Relavent Target Coordinates (mm39)*	*sgRNA sequence (5'-->3')*
G4 ESCs (XY, 129 / Sv x C57BL / 6 F1 hybrid)	DOI: 10.1073/pnas.0609277104	N/A	N/A	N/A	N/A	N/A
Dathe-Meis2	this paper	Wild type G4 ESC	Knock In	H11_KI_fwd ; H11_KI_rv	chr11: 3,195,448-3,195,467	GCTGATGGAACAGGTAACAA ; TTGTTACCTGTTCCATCAGC
Dathe-Tbx3	this paper	Wild type G4 ESC	Knock In	H11_KI_fwd H11_KI_fwd ; H11_KI_rv	chr11: 3,195,448-3,195,467	GCTGATGGAACAGGTAACAA ; TTGTTACCTGTTCCATCAGC

Supplementary Table 2.

Oligonucleotides. This table contains oligonucleotide sequences used for expression construct cloning, ESC genotyping and sgRNA sequences for CRISPR-mediated knock-in at H11 locus and WISH experiments.

Name	Sequence (5'-->3')	Purpose
H11_Bmp2_h_er_FW	GCTGAAGCTGATGGAACAgcGCCATGGCATTAATCAGACA	Amplify Bmp2-Dathe enhancer for Gibson assembly
Bmp2_h_er_Hsp68_RV	ggctgctcagtttggatgttTTCAGCACACCGTGCTTATC	
Bmp2_h_er_Hsp68_FW	GATAAGCACGGTGTGCTGAAaacatccaaactgagcagcc	Amplify Hsp68 promoter for Gibson assembly
Hsp68_meis2_RV	ATCGTACCTTTGCGCCATcagtctGGCGCCGCGCTCTGCT	
Hsp68_Meis2_FW	AGCAGAGCGCGGCGCCagactgATGGCGCAAAGGTACGAT	Amplification of backbone and cp Meis2 cDNA
H11_Bmp2_h_er_RV	TGTCTGATTAATGCCATGGCgcTGTTCCATCAGCTTCAGC	
cpTbx3_Dathe_FW	CCTTCCAGAAGCAGAGCGCGGCGCCGAGTGGATGAACCTCTCCAT	Amplification of cp Tbx3 cDNA
cpTbx3_Dathe_RV	TCTTGCTGATCATGATTAGTGTTTGgatccagacatgataagata	
cpTbx3_Dathe_BB_FW	tatcttatcatgtctggatcCAAACACTAATCATGATCAGCAAGA	Amplification of Hsp68 H11 KI backbone from Meis2 construct
cpTbx3_Dathe_BB_RV	ATGGAGAGGTTCATCCACTCGGCGCCGCGCTCTGCTTCTGGAAGG	
Gen_Chr13_qPCR_F	GGGAGCTGACACCACTATTTAC	qPCR genotyping
Gen_Chr13_qPCR_R	ACATTAAACCCTGGGGGAAG	
qPCR_cpMeis2_Dathe_FW	TGGTTCCATCCAGAGACAAGC	qPCR genotyping
qPCR_cpMeis2_Dathe_RV	ATCGTACCTTTGCGCCATCA	
qPCR_cpTbx3_Dathe_FW	TCCAGAGACAAGCGAAGACA	qPCR genotyping
qPCR_cpTbx3_Dathe_RV	GAGGTAGGAACGGATGGTAGG	
GenoES-H11-3HA-FW	TTCACTGCATTCTAGTTGTGGTTTG	genotyping 3'HA
GenoES-H11-3HA-RV	GTTGGCAGTTTTGGCCAGTT	
GenoES-H11-5HA-FW	GTTAGGCTTGTGTCAACTGTTTG	genotyping 5'HA
GenoES_DatheEn_RV	CCTTTCTGGTCATTGAAAGGCAC	
WISH_Hsp68-cpMeis2_FW	TCTGGTTCCATCCAGAGACAAG	Generation of DEG-labelled WISH probe
WISH_Hsp68-cpMeis2_RV	ATGGTCAGCGAGGTTTGTGG	

Supplementary Table 3.

Recombinant DNA. This table contains recombinant DNA used for cloning of CRISPR guide vectors and expression constructs.

Recombinant DNA	***Source***	***Identifier***
pSpCas9(BB)-2A-Puro (PX459) V2.0 vector	Addgene	62988
H11-back-pTwist+Amp+High+Copy	Twist Bioscience	
cp-Meis2-cDNA	Twist Bioscience	
cp-Tbx3-pUC-GW-Amp	GeneWiz, Azenta Life Science	
H11-Hsp68-LacZ	N/A	
